# Reactive Attachment Disorder and Disinhibited Social Engagement Disorder in School-Aged Foster Children - A Confirmatory Approach to Dimensional Measures

**DOI:** 10.1007/s10802-015-0045-4

**Published:** 2015-07-02

**Authors:** Stine Lehmann, Kyrre Breivik, Einar R. Heiervang, Toril Havik, Odd E. Havik

**Affiliations:** Department of Clinical Psychology, Faculty of Psychology, University of Bergen, Bergen, Norway; Regional Office for Children, Youth and Family Affairs, Region South, Tønsberg, Norway; Institute of Clinical Medicine, University of Oslo, Oslo, Norway; Division of Mental Health and Addiction, Oslo University Hospital, Oslo, Norway; Uni Research, Regional Centre for Child and Youth Mental Health and Child Welfare, Bergen, Norway

**Keywords:** Reactive attachment disorder, Disinhibited social engagement disorder, Foster children, Confirmatory factor analysis, Maltreatment

## Abstract

This study aimed to investigate the factor structure and external correlates of the constructs Reactive Attachment Disorder (RAD) and Disinhibited Social Engagement Disorder (DSED) from the Diagnostic and Statistical Manual of Mental Disorders (DSM-5). The following were addressed: First, do our data support the DSM-5 conceptualization of RAD/DSED as two separate constructs? Second, are RAD and DSED distinct from other well-established dimensions of child psychopathology? Third, what are the external correlates of RAD/DSED in this sample? The study sample included 122 foster children aged 6–10 years. Foster parents completed the Strengths and Difficulties Questionnaire (SDQ), and the RAD/DSED-scale from the Developmental and Well-Being Assessment. Child protection caseworkers completed a questionnaire regarding exposure to maltreatment and placement history. Confirmatory factor analysis (CFA) of the RAD/DSED items identified a good fit for a model with a two-factor structure, which is congruent with the DSM-5 definition of RAD and DSED. A new CFA model, which included the RAD and DSED factors together with the four problem factors of the SDQ (emotional, conduct, hyperactivity-inattention, and peer problems), also demonstrated a good fit with our data. RAD and DSED were associated with the SDQ Impact scale and help seeking behavior. This was partly explained by the SDQ externalizing and peer problem subscales. Our findings lend support for the DSM-5 conceptualization of RAD and DSED as separate dimensions of child psychopathology. Thus, the assessment of RAD and DSED provides information beyond other mental health problems.

The concept of attachment disorder is central in the description and understanding of social malfunctioning in institutionalized children and 1represents a major psychological etiological model that links early maltreatment to later psychopathology (Goldfarb 1945a, b; Tizard and Rees 1975). Attachment disorder is defined in terms of markedly disturbed and developmentally inappropriate social relatedness in most social contexts which begins before the age of 5 years, persists over time, and it is assumed to originate from very depriving and pathogenic care conditions (Rutter et al. 2009). Both the *International Classification of Diseases* (ICD-10; World Health Organization 1992) and the *Diagnostic and Statistical Manual of Mental Disorders* (DSM-5, American Psychiatric Association 2013) organize the symptoms of attachment disorder into two different but related disorders: an inhibited type termed Reactive Attachment Disorder (RAD) in both the ICD-10 and the DSM-5; and a disinhibited type termed Disinhibited Attachment Disorder (DAD) in the ICD-10 and Disinhibited Social Engagement Disorder (DSED) in the DSM-5. In this paper the term *attachment disorder* will be used to denote the general concept, while RAD and DSED refer to the two disorders as described in the DSM-5.

In the DSM-5, both disorders are described under the section Trauma- and Stressor-Related Disorders. Exposure to traumatic or stressful events is a shared criterion for disorders in this section. More specifically, social neglect and the absence of adequate caregiving during childhood are criteria for both RAD and DSED. The concept of attachment disorders has a long history in the study of psychopathology in school-aged children with an early and very deprived institutional background. Less is known regarding the structure and correlates of attachment disorders in foster children who have experienced neglect, but have not been institutionalized (Zeanah and Gleason 2010). In this study, we address three questions related to the understanding of attachment disorders: First, is there statistical support for the conceptualization of attachment disorders as two separate dimensions as described by the DSM-5, among school-aged foster children without institutional rearing? Second, are the dimensions of RAD and DSED distinguishable from other established dimensions of more common child psychopathology? Third, what are the correlates of RAD and DSED among characteristics of the placement history, exposure to risk factors in the biological family, functional impairment, and help-seeking behavior?

In a review of research covering the period from the introduction of attachment disorder in the DSM-IV in 1994 until the planning of the DSM-5, Zeanah and Gleason (2010) argued that despite the assumed similarities in etiology, research findings indicate that the two subtypes of attachment disorders should be understood as separate disorders: The inhibited subtype has been associated with depressed mood, whereas the disinhibited subtype has been associated with externalizing problems. Furthermore, a lack of social and emotional reciprocity and difficulties with emotion regulation are seen in the inhibited type, whereas the disinhibited type is characterized by a lack of developmentally appropriate discretion and restraint around strangers. Furthermore, children with the inhibited pattern appear to lack selective attachments and exhibit disinterest in interaction with adults, but they are responsive to enhanced caregiving. These considerations led to the current revision in the DSM-5 that classified the two patterns as separate disorders – RAD and DSED.

The main empirical support for this conceptualization of attachment disorder into RAD and DSED originates from two longitudinal studies on children raised in extremely deprived institutional contexts: the English and Romanian Adoptees Study (O’Connor et al. 1999) and the Bucharest Early Intervention Project (BEIP) (Smyke et al. 2002). Findings from the BEIP study support the two sub-patterns of attachment disorders as associated but distinct syndromes (Gleason et al. 2011). In a sample of 136 children (age range 6 to 54 months), both patterns demonstrated stability over 2 years and were distinguishable from more well-established disorders such as depression and externalizing disorders. The two syndromes contributed independently to functional impairment among the children in the study. Because the majority of empirical studies on attachment disorders is based on institutionalized children raised in extraordinary conditions in very deprived orphanages, caution is warranted regarding the generalization of these findings to children who have experienced maltreatment but who have not been raised in deprived institutions (Glowinski 2011).

In looked after children without institutional rearing, relatively strong associations between measures of attachment disorders and indicators of more general psychopathology have been reported (Kay and Green 2013; Millward et al. 2006). This may be rooted in a high prevalence of mental health problems and comorbidity in these samples, possibly inflating the associations. Together with findings that indicate high interrelations between the inhibited and the disinhibited syndromes (Zeanah et al. 2004), the question arises whether these behavioral patterns are best seen as distinct constructs that comprise two independent disorders, or if they should be understood as a part of a common pathway for serious pathology that hinders children’s abilities to relate socially.

Another central question is whether other harmful care conditions in addition to institutional rearing, could result in the same developmental pathology as seen in these samples from extreme child populations. In a U.S. study, DSM-IV RAD was identified in 38 % of maltreated foster children 10–47 months of age (*N* = 94), based on interviews with the clinicians treating the children. Using the ICD-10 criteria, findings indicated that 35 % had the inhibited type, 22 % had the disinhibited type, and 17 % had a mixed version (Zeanah et al. 2004). The inhibited and disinhibited types from ICD-10 were not related to gender, ethnicity, or time in foster care. Mental disorders in biological mothers were associated with both inhibited and disinhibited patterns, whereas mothers’ substance abuse was associated only with the disinhibited type.

Attachment disorders have also been identified in older maltreated children (Kay and Green 2013; Kočovská et al. 2012; Millward et al. 2006; Minnis et al. 2002, 2009). We previously reported a point prevalence of 19 % for DSM-IV RAD in 6–12 year-old foster children (*N* = 279), assessed with the Developmental and Well-Being Assessment (DAWBA) (Goodman et al. 2000) diagnostic interview (Lehmann et al. 2013). Here, having a RAD diagnosis was associated with more exposure to violence in biological family and more previous placements. These findings suggest that the constructs of RAD and DSED may also be relevant for school aged non-institutionalized children with a history of maltreatment in their biological family. The same study reported high comorbidity between attachment disorders and other mental disorders (Lehmann et al. 2013). This is consistent with reports of high correlations between attachment disorder scales and general measures of child psychopathology, such as the Strengths and Difficulties Questionnaire (SDQ) (Millward et al. 2006) and the Child Behavior Checklist (CBCL) (Kay and Green 2013). Therefore, the unique contribution of RAD/DSED in describing mental health problems beyond what is captured by other dimensions of mental health problems, should be examined.

In three studies of children without early institutional rearing, the factor structures of different attachment disorder symptom scales have been examined with principal component analyses (PCA) (Kay and Green 2013; Minnis et al. 2007; Oosterman et al. 2010). Only one study, using the Disturbances of Attachment Interview in a sample of foster children aged 2–7 years (*N* = 60), reported a two-factor solution congruent with an inhibited and a disinhibited dimension (Oosterman and Schuengel 2007). In a sample of 10–16 year-old looked-after children (*N* = 153), Kay and Green (2013) identified four factors in the 24-item version of the attachment disorder scale of the DAWBA interview: disinhibited indiscriminate, attention seeking, superficial relationships, and unpredictability. They reported an association between disinhibited symptoms and peer relationship problems, in line with the findings from the BEIP study reported by Gleason et al. (2011). Kay and Green (2013) also reported associations between the DAWBA subscales derived from the PCA analyses and CBCL subscales. The third study was conducted in a large community sample of 8-year-old twins (*N* = 13,472) (Minnis et al. 2007). Using an 18-item version of the Relationship Problems Questionnaire, four factors of relationship problems were identified, one of them resembling disinhibited attachment behavior.

Together, these studies yielded inconclusive results regarding the factor structure of attachment disorders in non-institutionalized children. One should note however, that all three studies used PCA as their analytic approach. PCA is based on a formative indicator model which assumes causal indicators that are measured without error. Thus, PCA is not optimal for identifying latent common factors that are believed to determine imperfectly measured items. Here, exploratory factor analysis and confirmatory factor analysis (CFA) with focus on common factors should be applied instead (Markus and Borsboom 2013; Schmitt 2011).

In a recent study, a modified 10-item version of the Relationship Problems Questionnaire was used in a clinical sample of children aged 5–10 years (*N* = 152) (Vervoort et al. 2013). Here, exploratory and confirmatory factor analyses supported a two-factor structure that corresponded to the inhibited and disinhibited patterns, respectively. The generalizability of these findings needs to be assessed, as their sample comprised mainly boys (87 %) with severe mental health problems attending special education schools. The family care conditions for these children were also not reported. The findings in the Vervoort study did not address the uniqueness of these attachment problems. That is, would the inhibited and the disinhibited dimensions still be identified as two unique factors when measures of more common dimensions of child psychopathology are included in the CFA?

In the present study, we examine the validity of the construct of attachment disorder for school-aged, non-institutionalized foster children. More specifically, is the factor structure of attachment disorders as assessed with the DAWBA RAD/DSED scale (Goodman et al. 2000), consistent with the DSM-5 operationalization of two separate patterns of RAD and DSED? Alternatively, should this inhibited /disinhibited behavior be considered as a more general expression of impaired social functioning reflecting the consequences of a wide range of child mental problems? With the use of CFA, we compare two alternative structural models of attachment disorder: Model 1 hypothesizes one general factor of attachment disorder, whereas Model 2 hypothesizes two factors consistent with the two patterns of RAD and DSED as defined in the DSM-5. In accordance with previous findings (Vervoort et al. 2013), we hypothesize a better fit for Model 2. We also investigate whether RAD and DSED are distinct from other dimensions of mental health problems using a third model that adds the four problem subscales of the SDQ to the RAD and DSED factors used in Model 2. Finally, we examine potential correlates of RAD and DSED, including indicators of neglect, maltreatment, placement history, functional impairment, and help-seeking behavior.

## Methods

### Measures

The DAWBA RAD/DSED scale is one of 17 sections in the parent version of the DAWBA diagnostic interview (Goodman et al. 2000; Heiervang et al. 2007; Meltzer et al. 2003). The 14-item DAWBA RAD/DSED scale used here is a further development from the Child and Adolescent Psychiatric Assessment-Reactive Attachment Disorder questionnaire (Minnis et al. 2009), and a previous 24-item DAWBA attachment disorder scale used in the Kay & Green study. This older 24 item version of the attachment disorders scale is not included in the standard version of the DAWBA anymore (Goodman, personal communication, February 13, 2014). All the 14 items in the current DAWBA version were part of the earlier 24-item version.

The DAWBA RAD/DSED scale may be completed by parents and caregivers for children up to 10 years of age. It comprises 14 questions that describe social behaviors that cause concern for carers. The items are rated on a three-point scale: *No* (0), *A little* (1), *A lot* (2). They are organized into a RAD subscale of five items, with score range 0–10 (e.g., “Does he avoid emotional closeness with adults he knows well?”), and a DSED subscale of nine items, with score range 0–18 (e.g., “Is he worryingly overfriendly with strangers?”).

The SDQ (Goodman 1997) is a 25-item mental health questionnaire for 4–16 year-olds that can be completed by parents, teachers, and as a self- report from age 11 years (Goodman et al. 1998). The SDQ consists of five subscales measuring prosocial behavior, peer problems, emotional symptoms, conduct problems and hyperactivity-inattention symptoms. Each subscale comprises five items rated on a 3-point scale (0-1-2), with a total subscale score range of 0–10. A Total Difficulties score is computed by summing the three symptom subscales and the Peer Problem subscale; resulting in a score range of 0–40. The two-page version of the SDQ also includes an Impact scale, for assessing distress to the child and interference of symptoms and problems with the child’s everyday life (Goodman 1999). The parent version of the Impact scale comprises five items and has a total score range of 0-10. The SDQ subscales have shown good internal consistency and good to excellent discriminative validity in the current population (Lehmann et al. 2014).

A 10-item Child Protection Questionnaire was developed for the current study, in order to obtain information from the child’s caseworker at the child protection services. In Norway, out-of-home placements are sanctioned by the county board and information used in the case for custody is later available to the child’s caseworker in municipal child protection service. The questionnaire asks about care conditions in the biological family, placement history, and contact with child and adolescent mental health services (CAMHS) or school psychology services. Regarding care conditions, the caseworker was asked about the following information from the case file: serious neglect, exposure to physical violence, witnessing physical violence, exposure to emotional abuse (threats, verbal punishment, harsh criticism, hostility), witnessing emotional abuse, parental rejection of the child, parental physical or mental disability, parental serious somatic or mental disorder, parental drug or alcohol abuse, and parental death. The child’s caseworker was asked if any of the above care conditions were present prior to the out-of-home placement.

### Ethics

The Regional Committee for Medical and Health Research Ethics, Western Norway, approved the study. The Ministry of Children, Equality and Social Inclusion provided exemption from confidentiality for the caseworkers and foster parents who participated in the study.

### Sampling and Procedure

In Norway, most children in long-term foster care live in private households, as group homes are rare. If there are other children in the family, these are most often siblings of the foster child, or the biological children of the foster parents. Data collection started on September 1 2011, and lasted until end of February 2012. Children were eligible if they were between 6 and 12 years of age and had lived in their current foster home for at least five months following legally mandated placement. The sample was recruited from the 63 municipalities served by the Southern Regional Office for Children, Youth and Family Affairs (Bufetat) in Norway. According to the Bufetat register, there were 391 eligible children in these municipalities. Informative letters were sent to the heads of each municipal child protection office. The office heads were asked to review the list of foster children from the regional register and to add potentially eligible children, if any, to the list. This identified 28 additional eligible children. Furthermore, 20 children from the regional register had been returned to their biological families, or had been adopted and were therefore no longer eligible. Another three children were deemed ineligible because of serious neurological disabilities. Thus, the final number of eligible children was 396.

The municipal caseworker of each child was contacted by postal mail with information letters about the study, asking them to complete and return the Child Protection Questionnaire for the child/children of their responsibility. The Child Protection Questionnaire takes about 10–20 min to complete. The caseworkers were not offered any compensation for participating.

Foster parents of the 396 eligible children received a postal letter with detailed information about the study and instructions on how to complete the SDQ and DAWBA measures online from home. Consenting foster parents logged on to a secure website, where they first completed the SDQ for the child before going on to the DAWBA interview. Foster parents were asked to answer all the 17 sections of the DAWBA, covering relevant DSM-IV disorders. While the SDQ normally takes about 10 min to complete, the full DAWBA may take up to several hours depending on the amount of problems reported. The DAWBA RAD/DSED scale usually takes only around 5 min to complete. Foster parents were not offered compensation for the participation in the study.

### Study Sample

Of the 396 eligible children, SDQ and DAWBA completed by foster parents were obtained for 223 children (56.3 % response rate). Of these, 198 children were 10 years or younger. All foster parents did not complete every section of the DAWBA. A completed DAWBA RAD/DSED section was only available for 122 of the 198 children. There were no significant differences between children with (*n* = 122) and without (*n* = 76) this section completed, either regarding age, gender, or SDQ scores. In this final study sample of 122 children, a completed Child Protection Questionnaire was available for 92 children (75.4 %).

### Analytical Procedure

Due to features of the online administration system, there were no missing SDQ data or DAWBA RAD/DSED data for the study sample of 122 children. Frequency distributions and correlations were analyzed with the IBM SPSS Statistics for Windows, Version 22.0. Mean scale scores were computed by dividing the sum score of each scale by the number of items in the scale.

Confirmatory Factor Analysis (CFA) was performed using Mplus 7.1. (L. K. Muthén and Muthén 2012). The CFA models were estimated using a robust weighted least squares estimator with mean and variances adjusted (WLSMV), to account for the nature of the skewed categorical data (ordinal data with three options). All 14 DAWBA RAD/DSED items were treated as ordinal categorical variables in the models and their estimations.

To investigate our hypothesis of a two-factor structure consistent with the DSM-5 conceptualization, model fit for three alternative models were tested. Model 1 hypothesized one general factor for all 14 items (5 RAD and 9 DSED items). Model 2 hypothesized a two-factor solution based on the two DAWBA subscales and consistent with the DSM-5 distinction between RAD and DSED. Comparative tests of nested alternative models were based on the diff-test option (Brown 2006). In Model 3, we further tested the construct of attachment disorders. Here, Model 2 was expanded by including the four problem factors from the SDQ (Goodman 2001; He et al. 2013; Muris et al. 2003; Van Roy et al. 2008). Model 3 thus hypothesized six factors: RAD, DSED, Peer Problems, Emotional Symptoms, Conduct Problems, and Hyperactivity-Inattention Problems. For the SDQ Hyperactivity-Inattention factor, the item “Fidgety” was allowed to correlate with the item “Restless” based on previous findings in Norwegian samples (Ronning et al. 2004; Van Roy et al. 2008).

Finally, to explore the discriminant validity for the different psychopathology constructs, we estimated trait correlations between the six factors in Model 3. For these correlations, an upper limit of 0.85 has been suggested for the interpretation of factors as representing different traits (Brown 2006).

For the analysis of external correlates of RAD and DSED, the two subscale scores were treated as continuous variables, and associations were tested with age, gender, and selected placement variables. The variables Serious Neglect, Parental Mental Disorder and Parental Alcohol- or Drug Abuse were treated as dichotomous variables. Help seeking behavior was represented by two dichotomous variables: “School Psychology Services”; indicating previous assessment by psychologist at school, and “Contact with CAMHS”. For the variable “Contact with CAMHS” a confirming answer to any of three items (each coded no = 1, yes = 2) resulted in a positive value: currently receiving treatment in CAMHS; previously received treatment in CAMHS; or assessed by CAMHS. The continuous variable “Violence Exposure” (0–4) was an index comprising the sum of the following four dichotomous items (each coded no = 0, yes = 1): exposure to physical violence, witnessing domestic violence, exposure to emotional abuse (threats, hostility, rejection, or harsh verbal punishment), and witnessing emotional abuse towards family members (Lehmann et al. 2013). Due to the inclusion of binary variables in the analysis (i.e., gender, serious neglect, parent’s mental disorder, parent’s drug or alcohol abuse, contact with CAMHS, contact with school psychologist), correlation analyses were conducted with both parametric and non-parametric (Spearman’s rho) approaches. Findings were equivalent and results of parametric analyses are reported.

The unique effects of RAD and DSED on functional impairment, as measured with the SDQ Impact scale, were examined by multiple regression analyses in two different ways. First, the unique effects of the RAD and DSED scales on functional impairment were estimated when controlling for each of the SDQ scales Emotional Symptoms, Conduct Problems, Hyperactivity-Inattention, and Peer Problems one by one, in four separate multiple regression-analyses. Second, the RAD and the DSED scales and the four SDQ subscales were entered simultaneously in two separate multiple regression analyses with SDQ Impact scale as the dependent variable. The goal was to examine the effect of each of the five scales on the SDQ Impact scale when controlling for the other four subscales. The unique effect of RAD and DSED on help-seeking behavior was examined in logistic regression analyses controlling for each of the SDQ scales, one at a time.

## Results

Table 1 shows the characteristics of the study sample (*N* = 122). Table 2 shows the mean scale scores for SDQ Total Difficulties - and subscales, the DAWBA Total RAD/DSED and RAD-, DSED- subscales, as well as the maximum possible scale scores and Cronbach’s alpha for each scale. There was a significantly higher mean scale score on the DSED scale compared to the RAD scale, with a mean difference of 0.44 (*SD* 0.46, 95 % CI [0.53, 0.36], *t* = 10.68, *df* = 121, *p* < 0.001). One should note that whereas the DSED scale was rather normally distributed, the RAD scale was positively skewed with a possible floor effect. This implies that the behaviors measured by RAD scale are quite rare in this sample, compared to the behaviors measured by the DSED scale, possibly indicating more deviant behavior among children with an elevated score on the RAD scale.

**Table 1:** Characteristics of the study sample (*N* = 122)

	%	Mean	*SD*
Gender: Girls	57.0		
Age		8.00	1.54
Age at first placement (years)^a^		3.11	2.76
Age when placed in current foster home^a^		3.88	2.87
Years in current foster home^a^		3.84	2.87
Sum previous placements^a^		1.25	1.26
Violence exposure (range 0–4)^a*^	44.6	0.91	1.26
Serious neglect^a^	90.2		
Parent’s mental disorder^a^	57.6		
Parent’s drug/alcohol abuse^a^	56.5		
Contact with CAMHS^a^	52.2		
School psychology services^a^	41.0		

^a^ = information from Child Protection Questionnaire available for 92 children. * = percentage represent the proportion reporting any value higher than 0

*CAMHS* child and adolescent mental health services

**Table 2:** Mean scores with standard deviation, range, skewness, Kurtosis and Cronbach’s alpha for the four problem subscales and Impact scale in the Strengths and Difficulties Questionnaire (SDQ), the DAWBA RAD subscale, DSED subscale and total RAD/DSED Scale (*N* = 122)

Scale	N of items	Max score	Mean score	*SD*	Skewness	Kurtosis	α
SDQ Total Difficulties	20	40	0.75	0.41	0.14	−0.81	0.88
SDQ Emotional Symptoms	5	10	0.71	0.48	0.23	−0.82	0.69
SDQ Conduct Problems	5	10	0.58	0.48	0.66	−0.25	0.75
SDQ Hyperactivity-Inattention	5	10	1.19	0.58	−0.36	−0.87	0.82
SDQ Peer Problems	5	10	0.53	0.47	0.72	-0.14	0.70
SDQ Impact	5	10	0.51	0.53	0.69	−0.74	0.79
DAWBA RAD	5	10	0.35	0.37	1.26	1.46	0.60
DAWBA DSED	9	18	0.79	0.45	0.28	−0.60	0.82
DAWBA RAD/DSED	14	28	0.64	0.37	0.47	0.02	0.82

Mean score computed by dividing the sum score of each scale by the number items in the scale. Possible range of mean score: 0–2 for all scales. *SDQ* Strengths and Difficulties Questionnaire, *DAWBA* Developmental and Well-Being Assessment, *RAD* reactive attachment disorder, *DSED* disinhibited social engagement disorder

The fit of the three CFA models were evaluated according to the following standard fit indices (Jackson et al. 2009): chi-square, comparative fit index (CFI), and root mean square error of approximation (RMSEA). The recommended cut-offs for good fit are CFI ≥ 0.95, and RMSEA < 0.06 when using the WLSMV estimator (Yu and Muthen 2002). Model 1, which hypothesized an one-factor structure with an overall attachment disorder factor, did not yield a good fit with our data (*χ*^2^ = 130.59, *p* < 001, *df* = 77, RMSEA = 0.08, 95 % CI [0.05, 0.10], CFI = 0.92). Model 2, which hypothesized a two-factor structure that measured RAD and DSED behaviors separately, indicated a better fit (*χ*^2^ = 101.20, *df* = 76, *p* = 0.028, RMSEA 0.05, 95 % CI [0.02, 0.08], CFI = 0.96). As shown in Table 5, a chi-square test identified a significantly better model fit for the two-factor compared with the one-factor model.

Table 3 displays the means, *SD*s, response frequencies, and factor loadings on the two factors for each of the 14 items. One should note that items in the RAD scale had a very low prevalence of responses in the “A lot” category compared to the items in the DSED scale, consistent with the skewedness of this scale. All items had factor loadings that were good to excellent according the criteria proposed by Tabachnick and Fidell (2007).

**Table 3:** Confirmatory factor analysis of the DAWBA RAD and DSED Items (*N* = 122)

				Response frequencies %			Factor loadings			
Item #	Label	M	SD	No	A little	A lot	F1		F2	
	DAWBA RAD items						Est	SE	Est	SE
2	Resists being helped	0.42	0.64	66.4	25.4	8.2	0.72	0.11		
4	Avoids emotional closeness	0.23	0.48	79.5	18.0	2.5	0.44	0.12		
6	Gaze aversion	0.48	0.70	63.1	25.4	11.5	0.69	0.09		
10	Unpredictable in reunions	0.30	0.60	77.9	14.8	7.4	0.44	0.14		
13	On the lookout for danger	0.33	0.57	72.1	23.0	4.9	0.67	0.10		
	DAWBA DSED items									
1	Needs to be center of attention	1.59	0.67	9.8	21.3	68.9			0.88	0.07
3	Desperate for adult attention	1.15	0.79	24.6	36.1	39.3			0.79	0.06
4	Singles out adult in charge	1.16	0.75	21.3	41.0	37.7			0.59	0.08
5	Many shallow relationships	0.68	0.75	49.2	33.6	17.2			0.65	0.07
7	Overfriendly with strangers	0.57	0.72	55.7	31.1	13.1			0.73	0.06
8	Superficial affection	0.32	0.55	72.1	23.8	4.1			0.82	0.05
9	Wanders away	0.48	0.70	63.1	25.4	11.5			0.56	0.07
11	Intrusive questions	0.42	0.64	66.4	25.4	8.2			0.71	0.07
12	Hangs on to adults	0.77	0.80	45.9	31.1	23.0			0.59	0.08

*DAWBA* Developmental and Well-Being Assessment,, *RAD* reactive attachment disorder, *DSED* disinhibited social engagement disorder

The two factors from the 14 DAWBA RAD/DSED items were then included with the four problem subscales of the SDQ in a third model. As shown in Table 5, this model exhibited a good fit with the data (*χ*^2^ = 623.69, *df* = 511, *p* < 0.001, RMSEA 0.04, 95 % CI [0.03, 0.05], CFI 0.95). The item loadings on the six factors shown in Tables 4 and 5 were all moderate to strong (0.53–0.86) for the DSED items. The item loadings on the DSED factor did not change substantially from the two-factor model, shown in Table 3. For the RAD factor, Item 10 (“unpredictable in reunions”) and item 4 (“avoids emotional closeness”) had a somewhat weak, but still fair, loading of 0.44 in the two-factor model. In the six-factor model, the loading for item 4 dropped to 0.37.

**Table 4:** Confirmatory factor analysis of the RAD and DSED items and items from the four subscales-assessing child mental health problems in the Strengths and Difficulties Questionnaire (SDQ) (*N* = 122).

	F1		F2		F3		F4		F5		F6	
	Est	ES	Est	ES	Est	ES	Est	ES	Est	ES	Est	ES
SDQ emotional items												
Somatic	0.47	0.12										
Worries	0.62	0.08										
Unhappy	0.68	0.08										
Clingy	0.68	0.08										
Afraid	0.75	0.07										
SDQ conduct items												
Throws tantrums			0.81	0.06								
Obeys			0.77	0.05								
Fights			0.80	0.07								
Steals			0.72	0.08								
Lies			0.69	0.07								
SDQ hyperactivity-inattention items												
Restless					0.71	0.06						
Fidgety					0.78	0.06						
Distracted					0.82	0.06						
Reflect					0.83	0.06						
Attend					0.81	0.04						
SDQ peer problems items												
Is a loner							0.64	0.08				
Has friends							0.52	0.10				
Popular							0.81	0.07				
Bullied							0.69	0.08				
Better with adults than with children							0.83	0.06				
DAWBA RAD items												
Resists being helped									0.76	0.09		
Avoids emotional closeness									0.37	0.13		
Gaze aversion									0.66	0.08		
Unpredictable at reunion									0.63	0.11		
On the lookout for danger									0.53	0.10		
DAWBA DSED items												
Needs to be center of attention											0.86	0.07
Desperate for adult attention											0.79	0.06
Singles out adult in charge											0.53	0.09
Many shallow relationships											0.65	0.08
Overfriendly with strangers											0.64	0.07
Superficial affection											0.81	0.07
Wanders away											0.58	0.08
Intrusive questions											0.77	0.07
Hangs on to adults											0.68	0.08

*SDQ* Strengths and Difficulties Questionnaire, *DAWBA* Developmental and Well-Being Assessment, *RAD* reactive attachment disorder, *DSED* disinhibited social engagement disorder

**Table 5:** Model fit results from the confirmatory factor analyses of the three models

Model	Chi-square			RMSEA	95 % CI	CFI		Difftest		
	X^2^	df	*p*					X^2^	df	*p*
1	130.59	77	0.00	0.08	0.05–0.10	0.92				
2	101.20	76	0.03	0.**05**	0.02–0.08	0.**96**	Model 1 vs Model 2	19.33	1	0.00
3	623.69	511	0.00	0.**04**	0.03–0.05	0.**95**				

*Model 1*: one-factor model of DAWBA RAD/DSED items. *Model 2*: Two-factor model of RAD and DSED items. *Model 3*: Six-factor model of items in RAD, DSED, SDQ Emotional Symptoms, Conduct Problems, Hyperactivity-Inattention Problems and Peer Problems subscales

*Bold*: Met the recommended cut-offs for fit indices

Given the relative small sample size, great care was taken to check for convergence problems and inadmissible parameter estimates. Furthermore, for sensitivity analyses we tested the robustness of the results of the CFA analyses for the three models, by rerunning the analyses with a Bayesian estimator (with uninformative priors) as this estimator is less sensitive for small sample sizes (B. Muthén and Asparouhov 2012; van de Schoot et al. 2014). The fit of the CFA models when using the Bayesian estimator was assessed by the Posterior Predictive p-values (PPP), a direct measure of the true discrepancy between data and the predicted model (Gelman et al. 1996). A low PPP value (e.g., 0.01) indicates poor fit, while an excellent fitting model is expected to have a PPP value of approximately 0.50 (B. Muthén and Asparouhov 2012). In the present study we used PPP values less than 0.05 to indicate a poor fit (Zyphur and Oswald 2013).

Somewhat in contrast to findings when using the WLMSV estimator, both Model 1 and 2 had an acceptable fit (PPP > 0.05), when using the Bayes estimator. But Model 2 (PPP value 0.29, 95 % CI for the difference between the observed and replicated *χ*^2^ values [− 41.71, 57.72]) had a somewhat better fit than Model I (PPP value 0.23, 95 % CI for the difference between the observed and replicated *χ*^2^ values: [− 28.94, 66.78]), also when using this estimator. Model 3 had also an adequate fit to the data (PPP value 0.10, 95 % CI for the difference between the observed and replicated *χ*^2^ values: [−41.29, 179.58]) when using the Bayes estimator. In these analyses, the model parameter values (factor loadings etc.) were in all cases very similar to what was found when using the WLMSV estimator. In sum, the Bayesian estimation confirmed our findings with use of the WLMSV estimator.

Table 6 shows the trait correlations for the general CFA model with six factors. All factors were positively correlated (0.50–0.89). The results showed a rather strong inter-correlation between the RAD and the DSED factors (0.59). A Wald test of parameter constraints showed that the Conduct Problem factor correlated significantly stronger with the RAD factor than with the DSED factor (5.34, *df* = 1, *p* = 0.023).

**Table 6:** Correlations between latent factors for SDQ subscales and DAWBA RAD/DSED scales (*N* = 122)

Factor	F1	F2	F3	F4	F5	F6
F1 SDQ Emotional Symptoms	1.00					
F2 SDQ Conduct Problems	0.69	1.00				
F3 SDQ Hyperactivity-Inattention	0.73	0.89	1.00			
F4 SDQ Peer Problems	0.68	0.80	0.69	1.00		
F5 DAWBA RAD	0.57	0.79	0.77	0.62	1.00	
F6 DAWBA DSED	0.50	0.53	0.70	0.60	0.59	1.00

*SDQ* Strengths and Difficulties Questionnaire, *DAWBA* Developmental and Well-Being Assessment, *RAD* reactive attachment disorder, *DSED* disinhibited social engagement disorder

Table 7 shows the correlations between the RAD and DSED scale scores and selected external variables. The variable Serious Neglect was highly positively skewed, and therefore not included in this analysis. Male Gender and Parental Mental Disorders were associated with higher scores on the RAD scale, but the RAD and DSED scales were unrelated to the other indicators of aversive care conditions, as well as placement history. However, in the correlation analyses, both the RAD and DSED scales were associated with functional impairment, measured with the SDQ Impact scale, and help-seeking operationalized by Contact with School Psychology Service and CAMHS.

**Table 7:** Correlations between gender, age, impairment, care conditions before placement, placement history, and help seeking behavior, SDQ subscale scores and the DSED and RAD scale scores (*N* =122)

	DSED	RAD
	*r*	*r*
Gender	0.00	−0.18^a^*
Age	−0.03	0.05
Age at first placement (years)^#^	0.19	0.11
Age when placed in current foster home^#^	0.23*	0.16
Years in current foster home^#^	−0.08	−0.19
Sum previous placements^#^	−0.01	−0.04
Violence exposure^#^	0.11	0.20
Parent’s mental disorder^#^	−0.02	0.21*
Parent’s drug/alcohol abuse^#^	−0.05	−0.04
Contact with CAMHS	0.27**	0.31**
School psychology services	0.26**	0.21*
SDQ impact scale	0.31**	0.44**

^#^ = variables derived from the Child Protection Questionnaire: *n* = 92. Contact with *CAMHS* assessed and/or received treatment by Child and Adolescent Mental Health Services, *SDQ* Strengths and Difficulties Questionnaire, *RAD* reactive attachment disorder, *DSED* disinhibited social engagement disorder. ^a^ boy = 1, girl = 2. * = *p* < 0.05; ** = *p* < 0.01

As presented in Table 8, the associations between the RAD scale and functional impairment remained significant when controlling for three of the SDQ subscales (Emotional -, Hyperactivity - and Peer Problems), but not when controlling for Conduct Problems (*β* = 0.13, *p* = 0.102). Also, the association between RAD and functional impairment became insignificant (*β* = 0.03, *p* = 0.621) in the analysis including all five scales.

**Table 8:** Contribution of RAD and DSED scale scores on functional impairment after controlling for SDQ Subscale scores of emotional symptoms, conduct problems, hyperactivity-inattention and peer problems in four separate regression analyses; and contribution of each subscale score after controlling for all other subscale scores in a multiple regression analysis

		Carer rated level of functional impairment			
		DAWBA RAD / DSED controlled for one SDQ subscale at a time		All five subscales entered simultaneously	
DAWBA	SDQ	β	*p*	β	*p*
RAD *r* = 0.439				0.036	0.622
	Emotional Symptoms	0.29	0.000	0.098	0.024
	Conduct Problems	0.13	0.102	0.249	0.008
	Hyperactivity- Inattention	0.16	0.032	0.325	0.000
	Peer Problems	0.25	0.002	0.213	0.008
DSED *r* = 0.305				−0.170	0.023
	Emotional Symptoms	0.14	0.107	0.108	0.150
	Conduct Problems	0.05	0.472	0.251	0.005
	Hyperactivity-Inattention	−0.10	0.244	0.411	0.000
	Peer Problems	0.07	0.382	0.251	0.002

*SDQ* Strengths and Difficulties Questionnaire, *DAWBA* Developmental and Well-Being Assessment, *RAD* reactive attachment disorder, *DSED* disinhibited social engagement disorder. Functional Impairment is measured by the SDQ Impact scale

In the parallel analyses of the DSED scale, the association between DSED and functional impairment became insignificant when controlling for any of the four SDQ problem scales. However, in the analysis including all five scales, DSED had a significant negative association (*β* = −0.17, *p* = 0.023) with the SDQ Impact scale score. One should note that in the analyses entering all five scales together, the scales assessing more externalizing problems, (i.e., Peer Problems, Conduct Problems and Hyperactivity Problems); were all associated with functional impairment.

The associations between RAD/DSED and Contact with CAMHS and School Psychologist were investigated in binary logistic regression analyses with RAD/DSED scale scores as predictors. Both RAD (crude *OR* 1.48, 95 % CI [1.12, 1.96], *p* = 0.006) and DSED (crude *OR* 1.15, 95 % CI [1.03, 1.29], *p* = 0.011) were associated with increased probability of contact with CAMHS, also when controlling for SDQ Emotional- and Peer Problems subscales. This association did however not remain significant when controlling for SDQ Conduct- and Hyperactive subscales. Neither the RAD nor DSED subscales significantly predicted Contact with School Psychology Services. No interaction effects were found between the independent variables.

## Discussion

In this school-aged sample of foster children without an early institutional rearing, our findings support the DSM-5 categorization of RAD and DSED as two conceptually and statistically separate dimensions of psychopathology. The RAD and DSED dimensions are distinguishable from other common mental health problems, indicating that they capture variations in interpersonal psychopathology not accounted for by measures of more general psychopathology, i.e., the SDQ subscales. The similarity of the results when using the two estimators in the CFA indicates that the WLMSV estimator gave trustworthy results despite the relatively low sample size in the present study.

Our analytic approach to the factor structure of attachment disorders represents a development from the three previous studies of the internal structure of attachment disorders that used PCA (Kay and Green 2013; Minnis et al. 2007; Oosterman et al. 2010). The good model fit obtained in CFA procedures, suggests that the DAWBA RAD/DSED items measure patterns of behavior that correspond well with the construct of RAD and DSED in DSM-5. To a large extent, our findings of a two-factor structure in the CFA are consistent with the findings reported by Vervoort et al. (2013). Furthermore, the present results expand the findings of Veervort, by preserving a two-factor structure in a more exacting model including four major dimensions of child psychopathology. The good model fit supports the argument that the behavior patterns seen in attachment disorders are not merely cross-dimensional side effects of more common psychopathology, but constructs in their own right, and should be assessed accordingly in foster children.

Despite overall moderate to strong factor loadings for the two factors in model 2, item 4 (avoids emotional closeness) and item 10 (unpredictable at reunions) had rather low (but still acceptable) loadings on the RAD factor. This indicates that these two items do not distinguish particularly well between children with high versus low levels of inhibited behavior. Furthermore, these two items have a low frequency of confirming answers from foster parents (Table 3). It is possible that these items are less developmentally appropriate for school-aged children. Because our sample comprised the older end of the age range for the DAWBA RAD/DSED scale, the discriminative ability of these items should also be tested in younger samples.

Whereas the DSED scale was rather normally distributed, the RAD scale was positively skewed. This implies that the behaviors measured by the RAD scale are rare in this sample, compared to the behaviors measured by the DSED scale, and this may indicate more deviant relational behavior among children whose scores are elevated on the RAD scale. The finding that the conduct problem factor in SDQ had a higher trait correlation with the RAD factor than with the DSED factor, are in line with the interpretation that children with elevated scores on the RAD scale may be at higher risk of also having conduct problems.

The correlation between the RAD and DSED factors was strong (*r* = 0.60), but comparable to correlations with and between other dimensions of psychopathology assessed with the SDQ. The high inter-correlations between the six psychopathology factors in Model 3 are consistent with the high comorbidity in this sample (Lehmann et al. 2013), and correspond to the relatively high associations between measures of attachment disorders and indicators of more general psychopathology reported in previous studies (Kay and Green 2013; Millward et al. 2006). This high interconnectedness may not be present in community samples with lower prevalence rates and less comorbidity. The present findings may be relevant to the argument for the use of dimensional assessment scales for symptom patterns that allow for profiles of symptom scores that may cross diagnostic boundaries (Rutter 2011) in high-risk samples. Still, our results show that these factors are separable into six distinct factors, corresponding to established dimensions of pathology.

Our results provide support for the conceptualization of attachment problems in two separate constructs describing aberrant social relatedness. However, a key question is whether the two constructs identified in the present study really represent disordered attachment, or rather some other psychological or social problem. The importance of social relationships in psychological development and for the understanding of mental disorders is well recognized (Rutter et al. 2009). Nevertheless, the diagnostic criteria for attachment disorders have been criticized for focusing on aberrant social behaviors and not specifically on attachment behaviors (O’Connor and Zeanah 2003). It has been argued that the defining feature of attachment disorders is a disturbance in the child’s use of a primary caregiver as a source of safety and security. Studies of non-institutionalized children have led to the proposal of a distinction between “nonattachment” and “disordered attachment”; the latter referring to existing, but severely disturbed attachment relationships (Zeanah and Boris 2000). This distinction may be useful in framing the empirical findings of RAD and DSED behavior in both institutionalized children and in children reared in a context with a primary caregiver where the relationship poses danger and insecurity for the child.

Nevertheless, both higher age and the lack of institutional experience leave uncertainty about the validity of the concept of RAD and DSED in our sample. Also, given that the information about disordered attachment behavior are collected from foster parents, this behavior could be an expression of more general and broad pathology, caused by high comorbidity. However, if this was the case, one would then expect the third CFA model, where the RAD and DSED factors are analyzed together with four other different problem areas, to show substantial correlations between the RAD/DSED factors and the more established SDQ factors. Further, the model would present significant cross-loadings between the items of the RAD/DSED factors and the items of the SDQ factors. This was not the case in our sample, and this implies that that the concept of RAD and DSED are relevant for older foster children with a high prevalence of comorbidity.

A stronger trait-correlation between RAD and SDQ Conduct Problems, suggests a higher probability of developmental pathology for children with elevated RAD scores, relative to children with elevated DSED scores. However, we cannot rule out a negative effect of DSED that is mediated through concurrent internalizing or externalizing behavior.

In line with our findings of rather strong zero-order correlations between RAD and DSED behavior and functional impairment, the results also indicate positive associations between the two scales and use of mental health services. However, the multiple regression analyses clearly indicate that relations between the RAD and the DSED scales and help-seeking behavior and functional impairment seem to be explained by the associations between RAD /DSED behavior and externalizing and peer problem behaviors. These findings are in line with other studies indicating that externalizing behavior are more often detected, and receive adequate treatment (Chavira et al. 2004; Heiervang et al. 2007). Thus, it is possible that RAD and DSED behavior in children may be overlooked by carers in the presence of more externalizing problems. The negative association between DSED score and SDQ Impact score in the model including all SDQ scales is somewhat surprising. This result needs to be replicated in further studies to test its validity.

The lack of associations between external correlates and measures of RAD/DSED is not in accordance with our previous findings, where both violence exposure and the number of previous placements were associated with the a binary indicator of the presence of DSM-IV attachment disorder (Lehmann et al. 2013). This could be a result of differences in the operationalization of attachment disorders and the statistical approaches used in the two studies. In the currently study, we used continuous DSED and RAD scale scores, rather than a dichotomous diagnostic classification.

### Strengths and Limitations

The strength of this study lies in the use of structured, clinically relevant instruments in a large sample that is representative for children placed out of home due to maltreatment. Nonetheless, of the total sample of 198 children aged 6-10 years old, the foster parents completed the DAWBA RAD/DSED section for only 122 children. This may decrease the generalizability of our findings, by increased risk for non-response bias. However, the children with DAWBA RAD/DSED information did not differ from the children without a completed DAWBA RAD/DSED section in terms of age, gender or measures of mental health problems.

The size of our sample reduces the power of some statistical analyses. Our results regarding the external correlates may have been affected by our relatively small sample size and prevented us from demonstrating the associations identified in previous studies (Kay and Green 2013; Minnis et al. 2002). Therefore, our results should be replicated in a larger sample. However, great care was taken to control for whether the analyses caused inadmissible parameters (i.e., negative variances); and none were identified.

The use of SDQ yield rather broadly measured domains of child mental health problems. One might argue that more direct and detailed measures of central symptom dimensions would target the testing of the RAD/DSED constructs with more precision. Nevertheless, with relatively few items on each subscale, the good model fit for six factors in this study indicate that the SDQ subscales capture distinct and independent areas of pathology, and that they measure constructs different from that measured by the DAWBA RAD and DSED scales. This is of clinical relevance, as the SDQ is widely used as a screening instrument both in CAMHS and in the child protection services.

As indicated by the relatively low mean and standard deviation on the RAD scale scores in this study, the full range of RAD behaviors were probably not observed in the present sample. This may be due to low frequency of these relational problems in foster children, or low sensitivity for the DAWBA RAD scale. Further studies with similar samples and alternate measures of RAD/DSED are needed to evaluate this.

In this study, we did not have access to observational data. Therefore, we do not know the correspondence between the carer information and the child’s actual behavior. Nevertheless, moderate convergence has been identified between caregivers’ reports of inhibited behavior and observed attachment behavior with the caregiver through the Strange Situation procedure (Zeanah et al. 2005). Furthermore, a caregiver’s report on a child’s “willingness to go off with a stranger” has shown a substantial convergence with the “Stranger at the Door procedure” (Zeanah and Smyke 2008). These findings lend support to the use of survey data based on caregiver reports to measure RAD/DSED behavior in children.

There is also a risk of common rater bias, as the foster parents were the sole informants on mental health disorders in this study, both on SDQ and the DAWBA RAD/DSED scale. On the other hand, our measures of adverse childhood experiences and placement variables were independent of foster parent rating.

Information on adverse childhood experiences was collected from caseworkers based on the children’s case files. The advantage of this method is that all maltreatment reports in official records are legally evaluated and identification of the occurrence is not done by individuals that are involved in the research project, or are informed about the research aims. Nevertheless, it has been argued that the official records represent sources of information about factors leading to being “caught”, rather than being a source of exhaustive information of maltreatment itself (Cicchetti and Toth 2005). Further, these data represent broad and general categories of exposure to maltreatment. The content of these indicators will vary from case to case regarding the onset or duration of the different types of exposure. For future studies, a standardized questionnaire or interview administered to foster parents and/or to the children themselves may be a supplement to the information found in the case files of the child protection offices.

### Future Research Questions and Clinical Implications

Our report is consistent with an emerging literature showing that the concept of attachment disorders, originating from studies of extremely deprived children raised in orphanages, is also relevant to foster children without institutional rearing. Along with previous studies, our results strongly indicate that the constructs of RAD and DSED are valid and discrete dimensions of child malfunctioning. Further research in this area is needed, including longitudinal studies evaluating the predictive validity of RAD and DSED constructs regarding mental health outcomes in later developmental stages. Also needed are studies of intervention effects on RAD and DSED behaviors.

The current study indicates that the assessment of school-aged foster children should include not only screening of internalizing and externalizing problems (e.g., with the SDQ), but also assessment of their relational functioning. More specifically, clinical alertness is warranted for children scoring positively on the DAWBA – RAD/DSED scale.
